# Linkages between women’s empowerment, religion, marriage type, and uptake of antenatal care visits in 13 West African countries

**DOI:** 10.1371/journal.pgph.0000406

**Published:** 2023-06-20

**Authors:** Michael Nnachebe Onah, Roseline Chinwe Onah, Felix Ezema Onah

**Affiliations:** 1 Institute of Public Policy and Administration, Graduate School of Development, University of Central Asia, Bishkek, Kyrgyzstan; 2 Department of Public Administration and Local Government, Faculty of Social Sciences, University of Nigeria Nsukka, Nsukka, Enugu State, Nigeria; 3 Department of Economics, Caritas University, Amorji-Nike, Enugu State, Nigeria; PLOS: Public Library of Science, UNITED STATES

## Abstract

Characteristics which reflect a particular context and unique to individuals, households, and societies have been suggested to have an impact on the association between women’s empowerment and women’s well-being indicators. However, there is limited empirical evidence of this effect. We used access to antenatal care (ANC) to examine the main and interaction effects of women’s empowerment, religion, marriage type, and uptake of services in 13 West African countries. Data was extracted from Phase 6 and 7 of the Demographic and Health Survey, and we measured women’s empowerment using the survey-based women’s empowerment (SWPER) index for women’s empowerment in Africa. ANC visits as the outcome variable was analyzed as a count variable and the SWPER domains, religion, and marriage type were the key independent variables. We utilised ordinary least square (OLS) and Poisson regression models where appropriate to examine main and interaction effects and analyses were appropriately weighted and key control variables were applied. Statistical significance was established at 95% confidence interval. Findings suggest that being Muslim or in a polygynous household was consistently associated with disempowerment in social independence, attitude toward violence, and decision-making for women. Although less consistent, improved social independence and decision-making for women were associated with the probability of increased ANC visits. Polygyny and Islamic religion were negatively associated with increased number of ANC visits. Decision-making for Muslim women appear to increase the probability of increased number of ANC visits. Improving the conditions that contribute towards women’s disempowerment especially for Muslim women and to a lesser extent for those who reside in polygynous households is key towards better uptake of antenatal care services. Furthermore, targeting of interventions and polices that could empower women towards better access to health services should be tailored on existing contextual factors including religion and marriage type.

## Introduction

Uptake of antenatal care (ANC) services have been shown to improve the well-being of women, and women’s ability to access these services are also considered a key indicator of women’s autonomy and empowerment [1, 2]. The World Health Organization (WHO) recommends that pregnant women especially in low-and-middle income countries (LMICs) attend at least eight ANC visits to enable them access needed maternal information and healthcare that is crucial for their well-being and that of their fetus and new-born [3]. While numerous barriers to uptake of ANC services exist, lack of empowerment for women remain a key barrier to uptake [4, 5].

There is consensus that women’s empowerment improves the welfare of women and their households and hence, women should be targeted for empowerment initiatives [6–10]. However, there is no consensus yet on how empowerment should be measured and what empowerment indicators are more relevant for specific welfare and health indicators for women. Another area of agreement is the understanding that empowerment as a construct is heavily influenced by cultural contexts, prevailing norms, and attitudes [11–14]. We define contextual factors as ““…the cultural and social circumstances within which couple relationships exist” [15]. Kabeer [11] further argues that the examination of women’s empowerment ought to be done in a manner that recognizes entrenched social constructions that are part of women’s daily lives both within and outside their households. In addition, many studies that have applied the same empowerment measures among women in regions with similar economic circumstances have found varying linkages between women’s empowerment and health and welfare indicators further suggests that perhaps there are underlining contextual factors that are not captured or adequately analyzed in women’s empowerment literature [8, 16]. For this study, we examined two of these prevalent contextual factors, religion and marriage type.

The tenets of Christianity and Islam do not disempower women. Rather, they seek to liberate women and encourage women’s empowerment [17, 18]. However, religion and culture have entwined over the years and religion has become part of the cultural context of many countries especially in LIMCs [19]. Hence, in regions with similar traditional practices, religion will largely be part of those prevalent traditions. Christianity was introduced into West Africa as part of colonialism by missionaries from the global North and hence has the characteristics of practices that are prevalent in the global North including western education and less conservatism to women’s rights, which are now predominantly practiced by LIMCs colonised by the global North [20, 21]. The integration of Islam into cultural and traditional practices in West Africa has also resulted in more conservative Muslim societies that predominantly limit women’s autonomy and independence [22]. Hence, it is not surprising that evidence suggests that women in Christian majority countries have better welfare indicators than those who reside in Muslim majority countries [23, 24].

Polygyny presents a different marriage and household model that lends more credence to the cooperative conflict model of households against the unitary model [25, 26]. In polygynous households, different strategies including food consumption, socioeconomic status, and the number and sex composition of children are used by co-wives to exert power in relations between them and their male spouse [10, 27, 28]. Hence, the role of women’s empowerment within such households would vary significantly from that of other household types including monogamous households. Polygyny is embedded in some religion and also a prevalent traditional and cultural practice in many sub-Saharan African (SSA) countries [23]. While polygyny is an encouraged practice of the Islamic religion, in reality, polygyny is also practiced within Christianity since there are numerous Christian denominations that encourage polygyny and there are many Christian majority countries with high rates of polygyny (e.g., Kenya and South Africa) [29, 30]. On the other hand, there are many Muslim majority countries that predominantly practice monogamy (e.g., Senegal and Gambia) further suggesting that polygyny is not at the core of Muslim practice nor non-existing in Christianity. In SSA, the percentage of women living in polygynous households can range between 20% to 40% in West Africa [31] although polygyny is reducing in part due to the economic demands of larger households as proposed by Bercker, Grossbard, and Bergstrom [32–34]. Women in countries with high rates of polygyny differ from those who reside in countries that predominantly practice monogamy; fertility is higher, the age gap between men and women in a union is significantly higher, and mental health indicators are worse in polygynous countries [35]. This suggests that perhaps there are multilinked relationships between cultural, religious, and marriage practices which create norms and attitudes that act as barriers to women’s well-being and the uptake of key services including modern antenatal care.

West Africa is the region with the highest level of polygyny globally as the eight countries with the highest proportion of women living in polygynous households are in west Africa [36]. There is also high levels of the practice of Islamic religion in the region as eight of the 16 west African countries are Muslim majority countries and other non-Muslim majority countries have large Muslim populations [37]. West Africa has one of the lowest rates of antenatal care (ANC) visits in low-and middle-income countries (LMICs) [38]. In many west African countries, existing sociocultural norms and practices tend to be patriarchal and patrilineal and this tend to favour men thereby creating a gender divide that limit women’s empowerment [9, 39–41]. In west Africa, there are also existing gender gap in education, employment, wages, and other socioeconomic indicators [42]. These existing gender gaps in addition to prevalent contextual factors including norms and practices that dictate how women should behave within and outside households influence women’s ability to gain and express empowerment which in turn affect their uptake of health services including ANC [43–45]. However, these prevalent norms tend to have subtle uniqueness that are context-specific and might vary within and across countries [8]. This highlights the need to further investigate the linkages between these contextual factors, women’s empowerment, and uptake of health services.

The present study seeks to add to the limited literature by examining the link between women’s empowerment and uptake of ANC services in the context of prevailing sociocultural practices (marriage type and religion) in West Africa. ANC visit was chosen as the outcome of interest in the regression analyses since evidence suggests that religion and other sociocultural factors are also important determinants of service uptake [4, 46]. To answer this research objective, we first examined if religion and marriage type were associated with the three women’s empowerment domains (attitude toward violence, social independence, and decision making). Secondly, we examined if religion, marriage type and the three empowerment domains were associated with ANC uptake. Finally, we examined if the three women’s empowerment domains produced significant interaction effects with religion and marriage type towards ANC uptake. Women’s empowerment was measured using the Survey-based Women’s Empowerment (SWPER) index [47, 48], and we used data from 13 West African countries.

## Methods

### Ethics statement

The present study was conducted with secondary cross-sectional data from 13 west African countries using the latest rounds of the DHS in Phase 6 (2008–2013) and Phase 7 (2013–2018) survey rounds for which the ethics approvals for each country was obtained prior to the surveys. Hence, we did not require a new ethics submission and approval.

### Study design and data

The DHS cross-sectional data are publicly available and are deidentified and anonymized prior to publication. In total, 127,287 married women of reproductive age provided information on questions used to develop the SWPER index and 88,693 provided information on ANC visits. The actual number of complete observations in each regression model is provided in the tables. The 13 West African countries examined were Benin (BEN), Burkina Faso (BFA), Cote d’Ivoire (CIV), Gambia (GAB), Ghana (GHA), Guinea (GIN), Liberia (LBR), Mali (MLI), Niger (NER), Nigeria (NGA), Senegal (SEN), Sierra Leone (SLE), and Togo (TGO).

### Outcome variable

#### Antenatal care visit

The number of antenatal care (ANC) visits was examined as a count variable using Poisson regression.

### Key independent variables

#### Women’s empowerment domains

The three SWPER empowerment domains are continuous variables and were used as the key independent variables. These three domains examined women’s empowerment in *decision-making*, *attitude towards violence*, and *social independence*. Our hypothesis, which is in line with [47, 48] was that each domain might have different associations with antenatal care visits.

#### Overview of the Survey-based Women’s Empowerment (SWPER) index for women’s empowerment in Africa

The Survey-based Women’s Empowerment (SWPER) index was developed because of an identified need to have a more standardized index for measuring women’s empowerment in routine cross-national surveys in LMICs [47, 48]. The index was developed after a systematic review of the literature on measures of women’s empowerment [47, 48]. This review found that where available, indicators of women’s empowerment came mainly from reports that based their analysis on specific countries or surveys where information on gender equality and empowerment were selected and grouped arbitrarily, and weights to empowerment indicators were defined without a clear strategy. To address this identified gap in knowledge, Ewerling et al. [47] developed the SWPER tool using data from the DHS as an index that encompasses three well known domains of women’s empowerment (*attitude to violence*, *social independence*, and *decision-making*), which requires response both from women and men in the household. Table 1 describes variables included in the SWPER index. Negative values in the mean empowerment score in any of the domains of empowerment imply that women are less empowered than men and vice versa for positive values.

**Table 1:** Coding system for the variables included in the composition of the SWPER index.

DHS questions	Code or unit
*Attitude towards violence*	
Beating justified if wife goes out without telling husband	Justified = -1; don’t know = 0; not justified = 1
Beating justified if wife neglects the children	Justified = -1; don’t know = 0; not justified = 1
Beating justified if wife argues with husband	Justified = -1; don’t know = 0; not justified = 1
Beating justified if wife refuses to have sex with husband	Justified = -1; don’t know = 0; not justified = 1
Beating justified if wife burns the food	Justified = -1; don’t know = 0; not justified = 1
*Social independence*	
Frequency of reading newspaper or magazine	Not at all = 0; <once a week = 1; ≥once a week = 2
Woman’s education	Years
Education difference: woman’s minus husband’s years of schooling	Years
Age difference: woman’s minus husband’s age	Years
Age at first cohabitation	Years
Age of respondent at 1st birth	Years
*Decision-making*	
Who usually decides on respondent’s health care	Husband or other alone = –1; joint or respondent alone = 1
Who usually decides on large household purchases	Husband or other alone = –1; joint or respondent alone = 1
Who usually decides on visits to family or relatives	Husband or other alone = –1; joint or respondent alone = 1

(source: Ewerling et al., 2020)

The first domain, attitudes towards violence, was dominated by questions related to the respondent’s opinion about whether wife-beating was justified or not in various scenarios. The second domain, social independence, included items related to education, information (frequency of reading newspaper or magazine), and age at first child’s birth and at first cohabitation. The differences between the woman respondent and her husband in terms of education and age also appeared in this domain, but with lower loadings. The third domain, decision-making, comprised questions about involvement in household decisions and, with a lower loading given to whether the respondent performed any paid work in the past 12 months. The SWPER enables within-country and between-country comparisons, and time trend analyses for African countries and can be calculated at the individual level. The methodology for creating the SWPER index can be found in Ewerling et al. [47, 48]. In total, 14 items were extracted from the DHS and principal component analysis factor loadings were used to construct the index.

### Religion

The prevalent religion based on the DHS were Christianity and Islam and was examined as dichotomous variable with ‘1’ denoting Christianity and ‘2’ Islam. The hypothesis is that religion would have varied main and interaction effects on the outcomes of interest. Three countries (Niger, Senegal, and Gambia) were excluded in the disaggregated regression analyses due to low heterogeneity in religion (i.e., much of the sample practiced Islam). While data on traditional religion as a third religious practice is collected as part of the DHS survey, the proportion of responses were low across countries hence we restricted our analyses to the two most prevalent religion.

### Marriage type

Two prevalent marriage types in West Africa were monogamy and polygyny. To generate this variable, we utilised two variables; “v502” (married/living with partner) and “v505” (number of other wives). Women with “0” other wives were considered to be in a monogamous marriage while women with “1 or more” other wives were considered to be in polygamous households. We examined marriage type as a binary variable (coded as ‘1’ Monogamy; ‘2’ Polygyny) and examined its main and interaction effects on uptake of ANC visits. This was based on our assumption that marriage type would have a different effect on the outcome of interest.

### Control variables

Since the SWPER uses a few socio-demographic variables (age, age at first sex and cohabitation, cohabitating status, and education level) to develop the index, we retained only key socio-demographic variables that were not included in the development of the index as controls. We assumed that these variables would have important confounding effects on the outcomes of interest. The control variables included were women’s employment status, urban-rural location, wealth index, household size, and region of residence.

### Empirical specification

In line with our hypothesis, we specified the following models and analyses were pooled and disaggregated per country.

Let *γ*_*i*_ be each SWPER domain estimated as:

$$\gamma_{i}=\beta_{0}+\beta_{1}Religion+\beta_{2}Marriage\_type+\beta_{3}C+\varepsilon$$ (1)

where *C* is a vector of other socio-demographic and economic control variables, *β*_1_ *and β*_2_ are the estimated parameters/parameter vectors, and ε is the error term.

To estimate uptake of ANC visits as outcome variable, let *Y*_*i*_ be the outcome variables and the Poisson model is estimate as:

$${In(Y}_{i})=\beta_{0}+\beta_{1}SWPER+\beta_{2}Religion+\beta_{3}Marriage\_type+\beta_{4}C+\varepsilon$$ (2)

where *ln*(*Y*_*i*_) is the log of outcome variable (ANC visits), *C* is a vector of other socio-demographic and economic control variables, *β*_1_, *β*_2_ *and β*_3_ are the estimated parameters/parameter vectors, and ε is the error term.

Estimating the interaction effect of religion and marriage type:

$${In(Y}_{i})=\beta_{0}+\beta_{1}SWPER+\beta_{2}Religion+\beta_{3}Marriage\_type+\beta_{4}C+\beta_{5}(SWPER\times Religion)+\varepsilon$$ (3)

$$In\;(Y_{i})=\beta_{0}+\beta_{1}SWPER+\beta_{2}Religion+\beta_{3}Marriage\_type+\beta_{4}C+\beta_{6}(SWPER\times Marriage\_type)+\varepsilon$$ (4)

where *β*_1_, *β*_2_, *β*_3_, *and β*_4_ and ε are as above. The relationship between women’s empowerment and ANC visit is given by *β*_1_. The impact of empowerment is the sum of the coefficients of the empowerment variables and the coefficient of the interaction term with the dummy variables (religion: (*β*_1_+*β*_5_); marriage type (*β*_1_+*β*_6_)). If the nest of the test of the differential impact of women’s empowerment, represented by the coefficient of the interaction term (*β*_5_ *and β*_6_) is significantly different from zero, then this suggests that women’s empowerment has differential effects based on religion and marriage type.

Stata version 15.1 was used and medians, percentages, and SDs were used to describe the study sample, outcome measures, and the SWPER domains. The association between religion, marriage type, and the three SWPER domains were examined using ordinary least squares (OLS) regression. The main and interaction association effects between the SWPER domains, religion and marriage type were examined using marginal effects of Poisson regression models. All the regression models were adjusted for the effects of specified control variables, controlled for cluster and stratum sampling effects, and appropriate weights were applied during analysis. Statistical significance was established at 95% confidence interval and only significant associations in the pooled analyses are reported in text. To account for the effect of individual countries in the pooled analyses, dummy variables for each country were included.

## Results

### Sample characteristics

On average, 63% of women belonged to monogamous households, and 61% belonged to Muslim households. Women’s education were about 2.9 years and husband’s education were about 4.2 years. Women had an average age of 31.4 years and husbands’ age were about 41.6 on average. Sixty-seven percent of households were in rural areas and the average household size was about 8. In total, 43% of households were in the lowest two socioeconomic groups based on their wealth index and 68% of women were currently employed. Further disaggregation of the sample characteristics can be found in S1 and S2 Tables.

### Antenatal care visits

**Fig 1:**
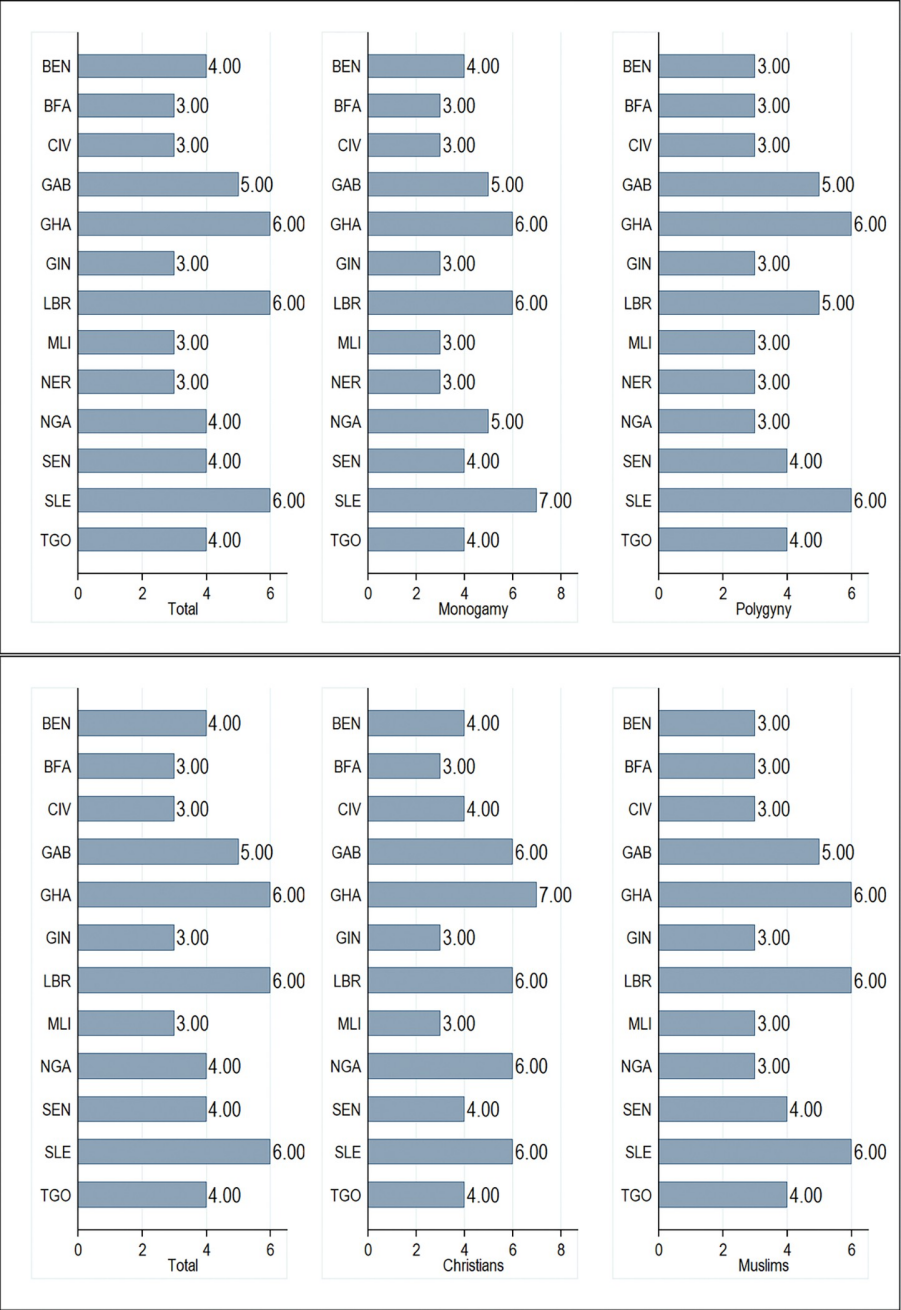
Median number of antenatal care visits by marriage type and religion.

In total, the median number of ANC visits was 4 for women during their last birth. Ghana, Liberia, and Sierra Leone reported the highest median number of ANC visits (6 visits) while Burkina Faso, Cote d’Ivoire, Guinea, Mali, and Niger recorded the lowest median number of visits (3 visits). Women who identified as Christians reported higher median number of ANC visits relative to those who identified as Muslims (5 vs 4 visits). Women who reported to live in a monogamous household reported higher median number of ANC visits relative to those who lived in polygynous households (4 vs 3 visits) Further disaggregation of mean ANC use by religion and marriage type for each country can be found in Fig 1.

### Mean empowerment scores for SWPER domains

**Fig 2:**
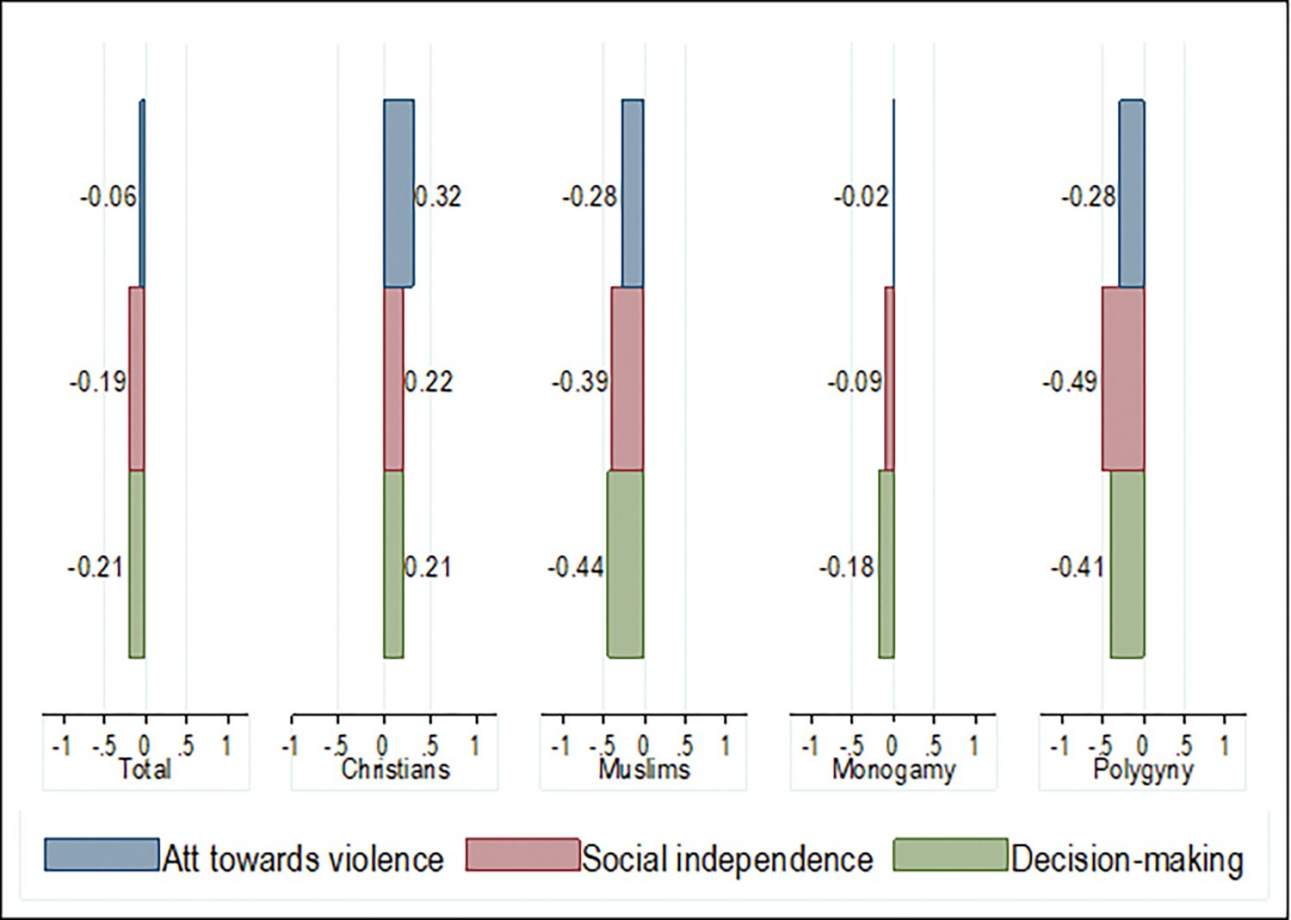
Mean SWPER scores across the three domains by religion and marriage type–pooled estimates.

**Fig 3:**
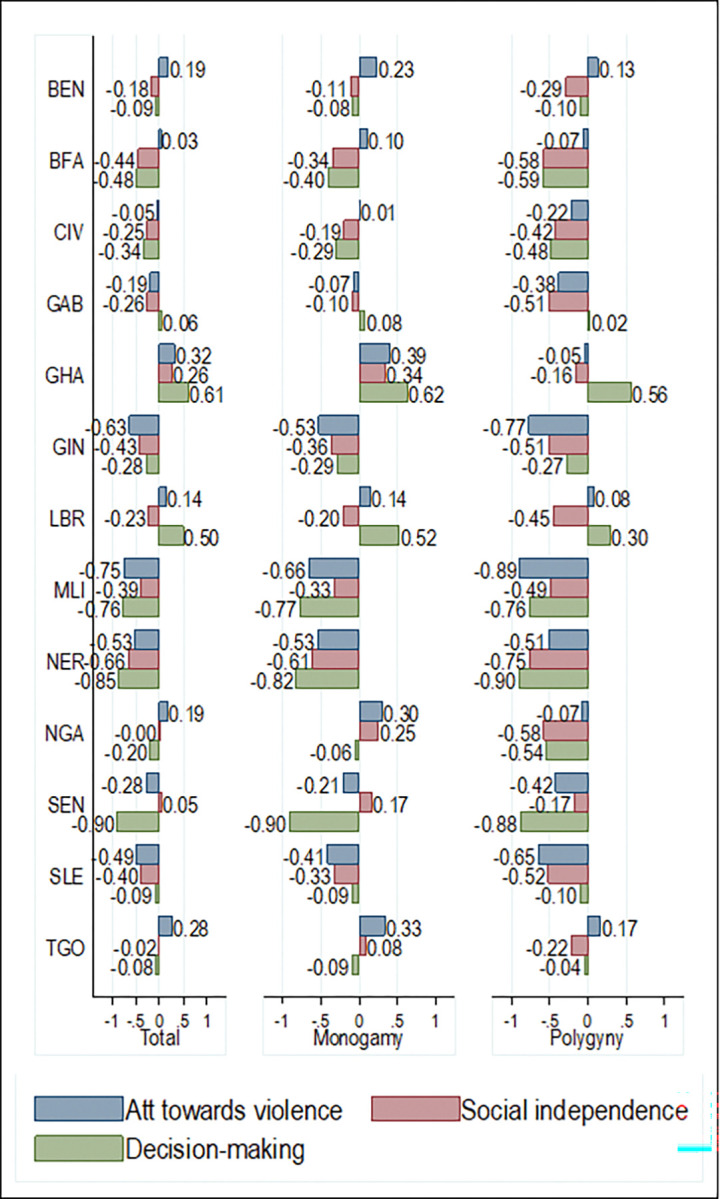
Country performance across the SWPER domains by marriage type.

**Fig 4:**
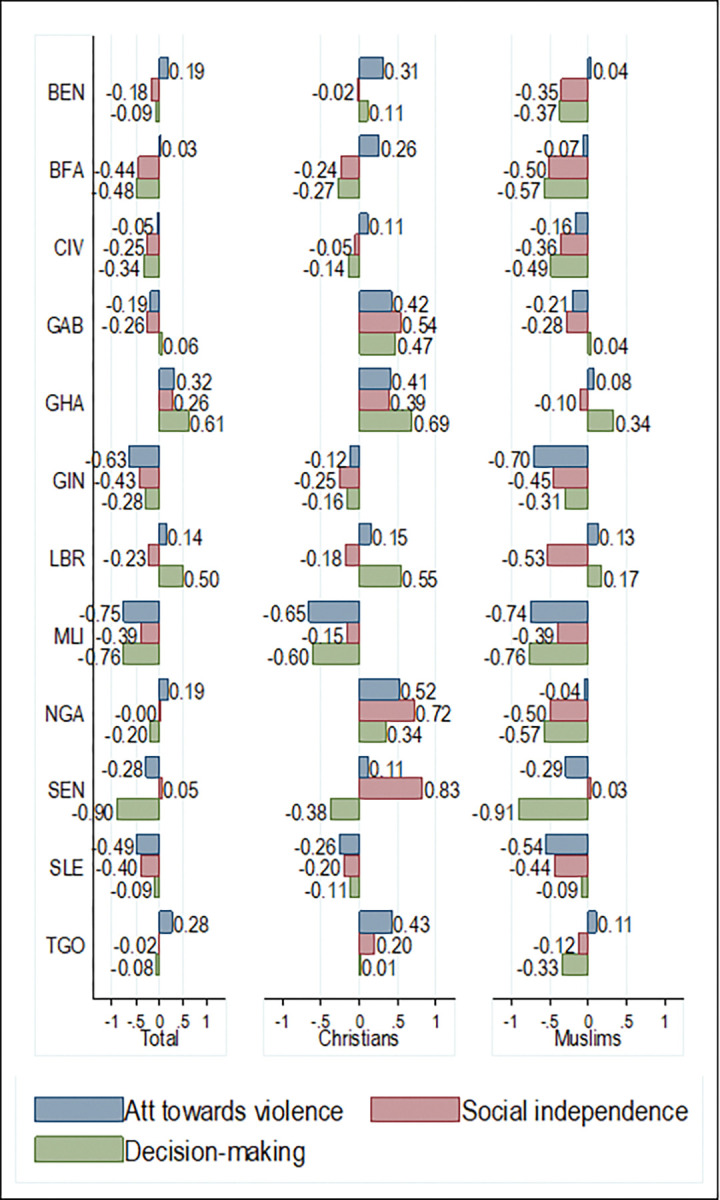
Country performance across the SWPER domains by religion.

In general, women were predominantly disempowered in West Africa across the three SWPER domains. Women scored -0.06 SD for attitude towards violence, -0.19 SD for social independence, and -0.21 SD for decision-making. Further, women who identified as Christians were moderately empowered across the three SWPER domains while women who identified as Muslims were disempowered across the three empowerment domains. Women who identified as Christians scored 0.32 SD for attitude to violence (-0.28 for Muslims), 0.22 SD for social independence (-0.39 for Muslims), and 0.21 SD for decision-making (-0.44 for Muslims). Women who belonged to monogamous and polygynous households were disempowered in their attitude towards violence (-0.02 and -0.278 respectively), social independence (-0.09 and -0.49 respectively), and decision-making (-0.18 and -0.41 respectively) although the magnitude of disempowerment was larger for women in polygynous households (Fig 2). These changes in the magnitude and direction of empowerment can also be observed in the disaggregated analyses across countries (Figs 3 and 4).

### Association between religion and marriage type, and the SWPER domains

**Fig 5:**
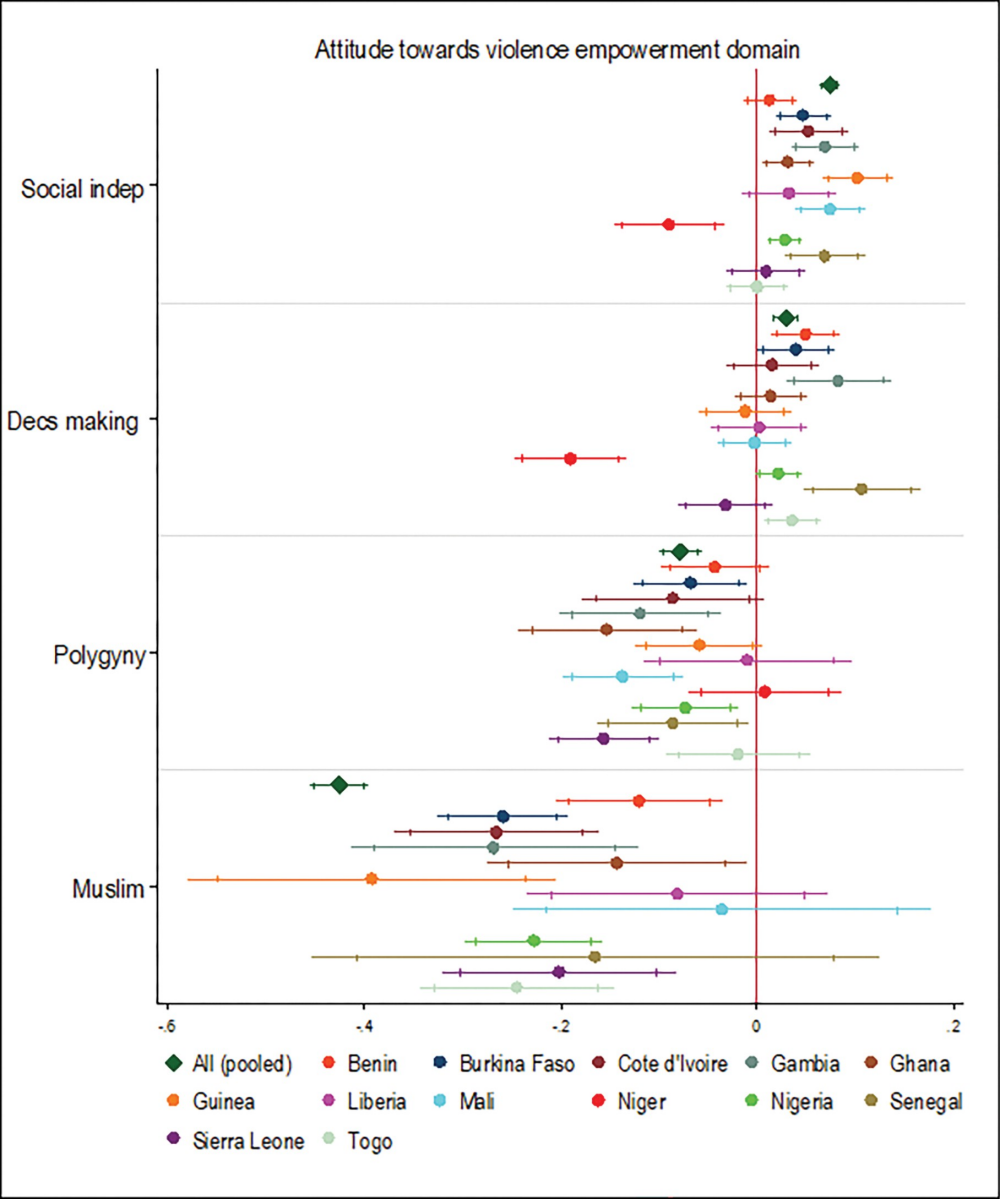
Association between religion, marriage type, and attitude towards violence empowerment domain. Note: Country dummy variable included in pooled analyses; economic and demographic control variables included in each model; spikes indicate 90% and 95% confidence intervals.

Belonging to polygynous households for women was associated with a 0.18 reduction in the SD scores for attitude to violence relative to women who belonged in monogamous households. This effect was statistically significant in the pooled analyses and in seven of the 13 West African countries examined (Fig 5). Also, being a Muslim was associated with a 0.43 reduction in the SD scores for attitude towards violence relative to being a Christian for women. This effect was statistically significant in the pooled analysis and in nine of the 12 West African countries examined.

**Fig 6:**
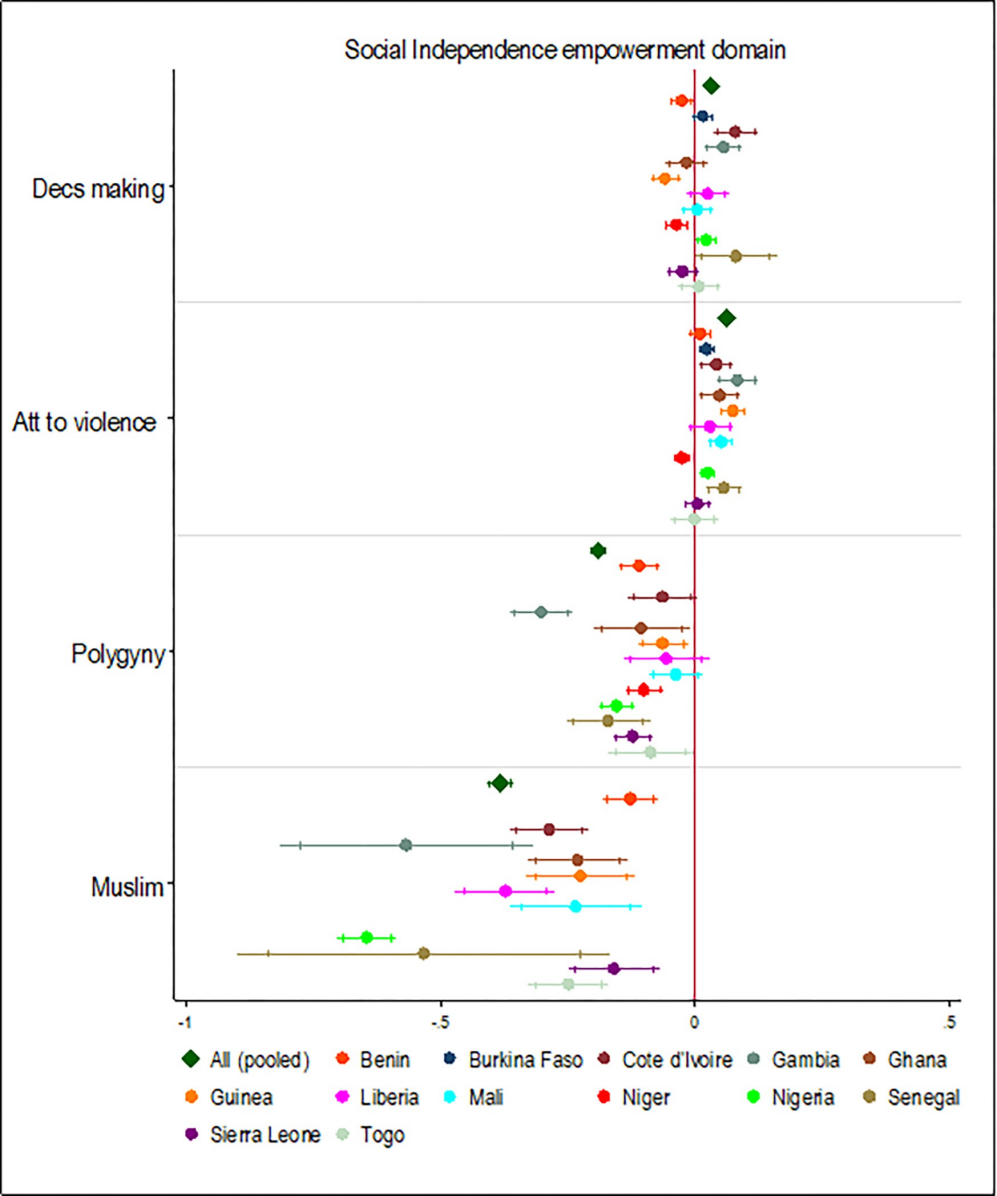
Association between religion, marriage type, and social independence empowerment domain. Note: Country dummy variable included in pooled analyses; economic and demographic control variables included in each model; spikes indicate 90% and 95% confidence intervals.

Belonging to polygynous households for women was associated with a 0.19 reduction in social independence SD scores relative to women who were in monogamous households. This effect was statistically significant in the pooled analyses and in 11 of the 13 West African countries examined (Fig 6). Also, being a Muslim was associated with a 0.38 reduction in social independence SD scores relative to being a Christian. This effect was statistically significant in the pooled analysis and all the West African countries examined.

**Fig 7:**
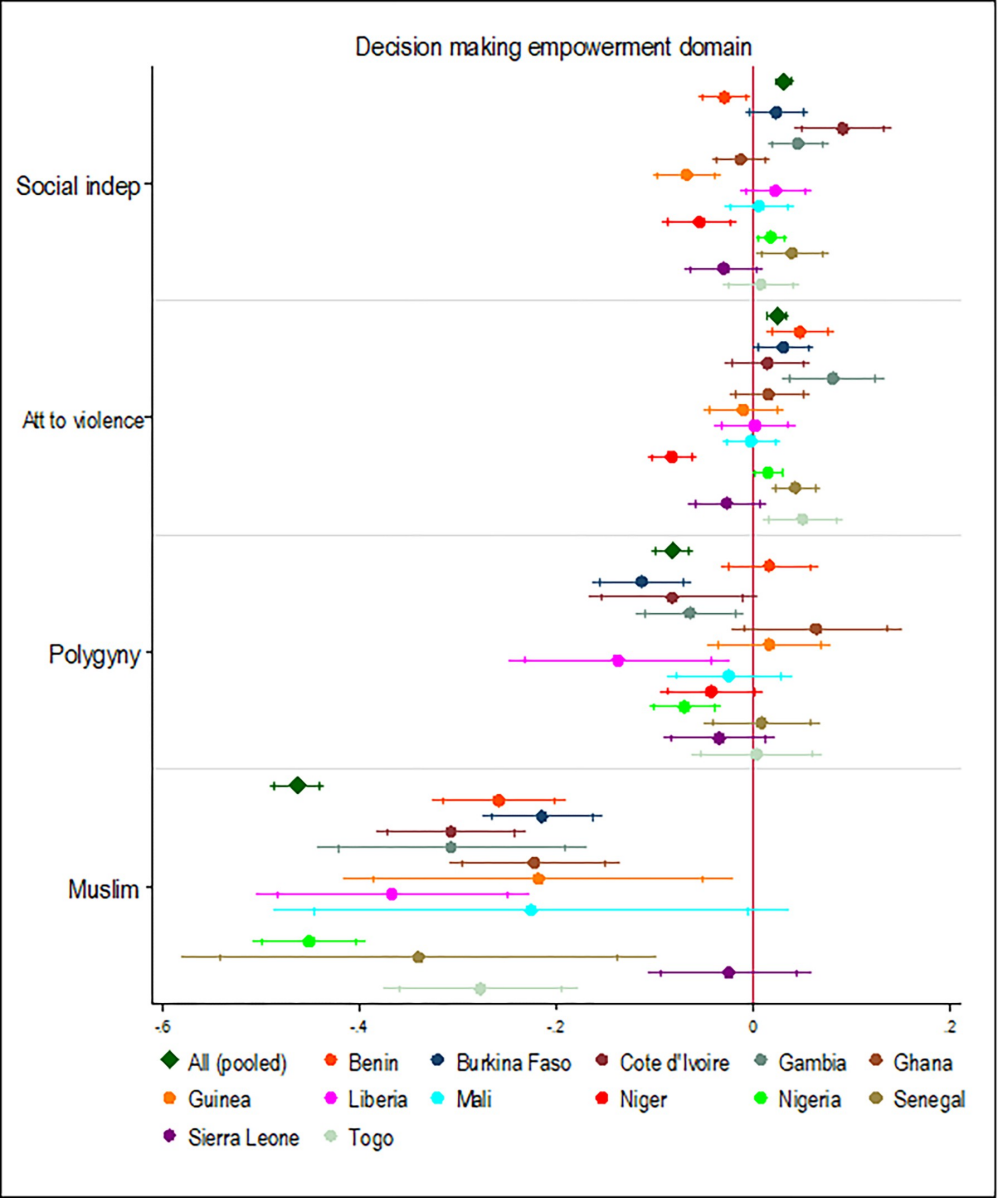
Association between religion, marriage type, and decision-making empowerment domain. Note: Country dummy variable included in pooled analyses; economic and demographic control variables included in each model; spikes indicate 90% and 95% confidence intervals.

Belonging to polygynous households for women was associated with a 0.08 reduction in the SD scores for decision-making and this effect was statistically significant in the pooled analyses and in five of the 13 West African countries examined (Fig 7). Also, being a Muslim was associated with a 0.49 reduction in decision-making SD scores relative to being a Christian. This effect was statistically significant in the pooled analysis and 11 of the 12 West African countries examined.

### Association between the SWPER domains, religion, and marriage type, and ANC visit

**Fig 8:**
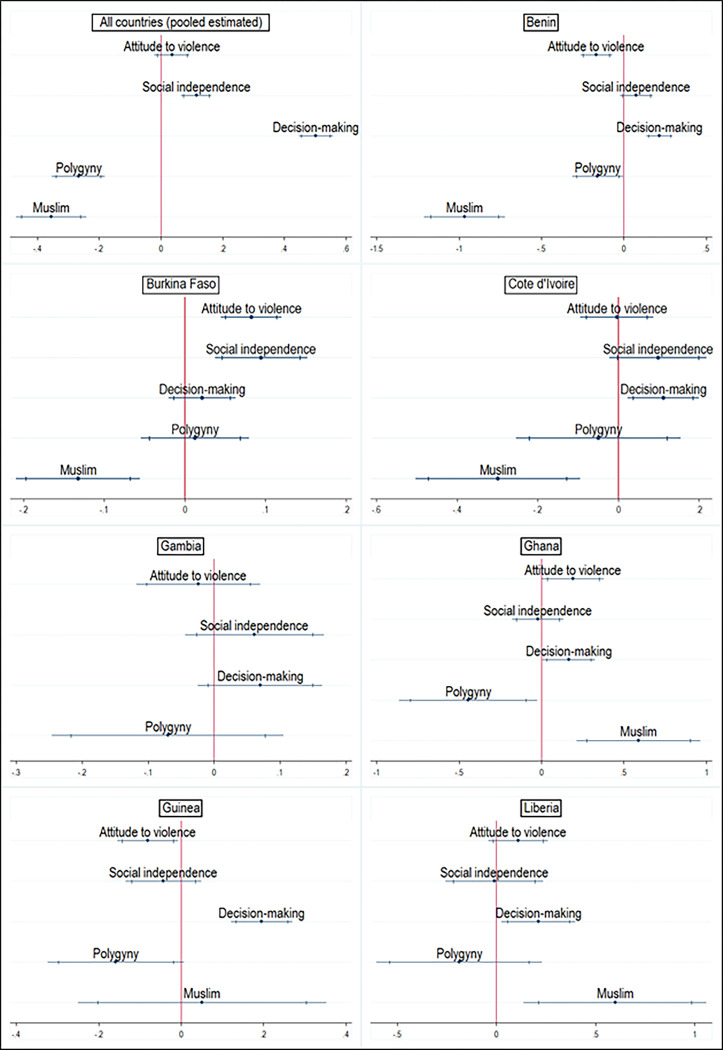
Association between SWPER domains, religion, and marriage type, and ANC visit. Note: Country dummy variable included in pooled analyses; economic and demographic control variables included in each model; spikes indicate 90% and 95% confidence intervals.

A unit increase in in the SD of attitude towards independence scores was associated with a 3.6%-point increase in the likelihood of women attending an additional ANC visit in the pooled analysis. This significant association was also found in six (Benin, Burkina Faso, Ghana, Nigeria, Senegal, and Sierra Leone) of the 13 countries included in the study. A unit increase in the SD of social independence score was associated with a 11.4%-point increase in the likelihood of women attending an additional ANC service during their last pregnancy and this association was also found in all the 13 countries examined except Liberia, Niger, and Sierra Leone. A unit increase in the SD of decision-making score was associated with a 50.2%-point increase in the likelihood of women attending an additional ANC service during their last pregnancy in the pooled analysis and all the 13 countries examined except Burkina Faso, Gambia, and Mali, (Fig 8).

**Fig 9:**
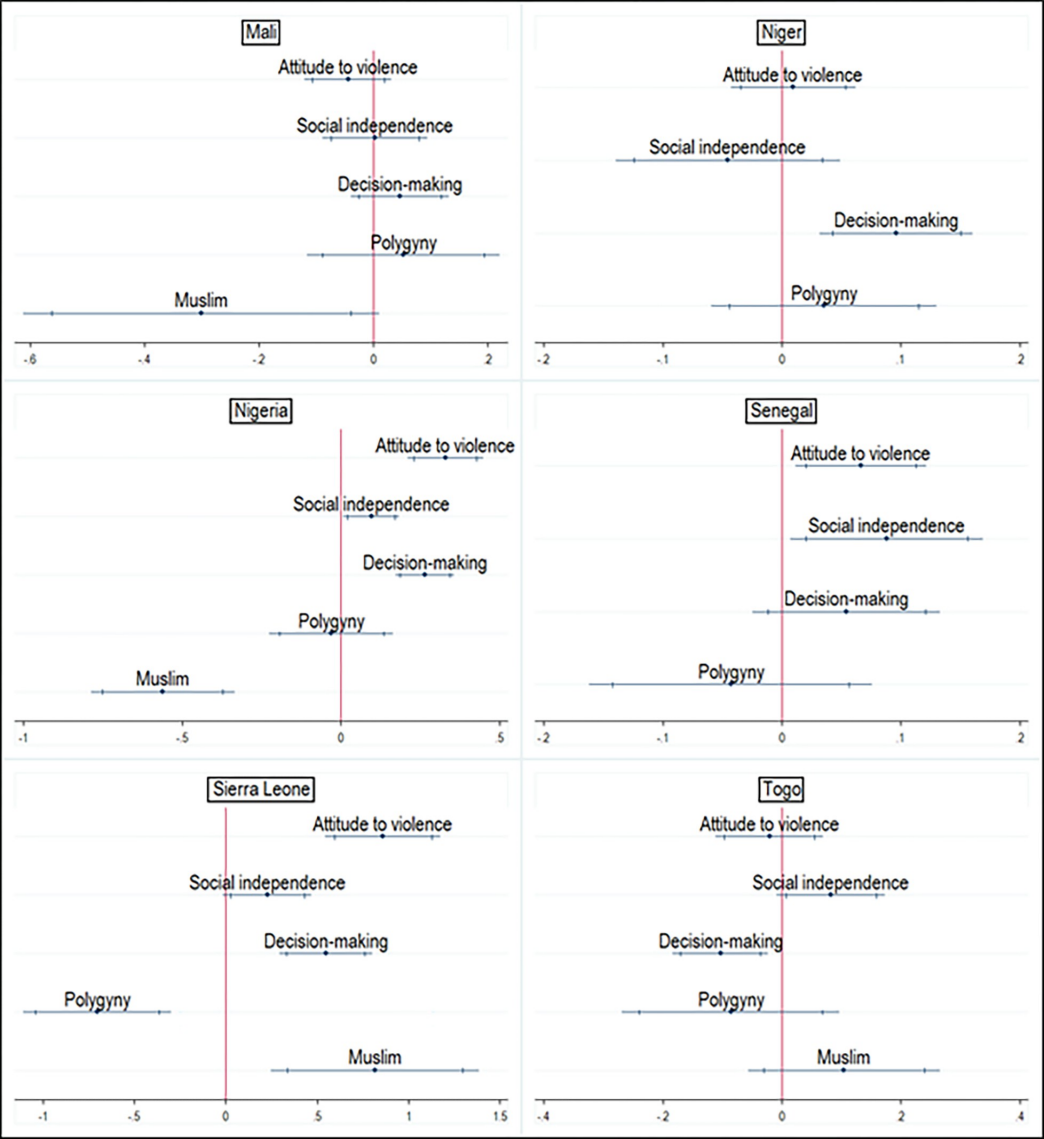
Association between SWPER domains, religion, and marriage type, and ANC visit (continued). Note: Country dummy variable included in pooled analyses; economic and demographic control variables included in each model; spikes indicate 90% and 95% confidence intervals.

Women who belonged to polygynous households were 26%-point less likely to attend more ANC services during their last pregnancy relative to those who belonged to monogamous households and this association was also found in only Benin, Guinea, Sierra Leone, and Togo. Relative to their Christian counterparts, women who identified as Muslims were about 36%-points less likely to attend more ANC services during their last pregnancy in the pooled analysis and in Benin, Burkina Faso, Cote d’Ivoire, and Nigeria. However, being a Muslim was associated with about 26%-point, 19%-point, and 46%-point increase in the likelihood of attending more ANC visits for women in Ghana, Liberia, and Sierra Leone, respectively (Figs 8 and 9).

### Interaction effects between the SWPER domains, religion, and marriage type

**Fig 10:**
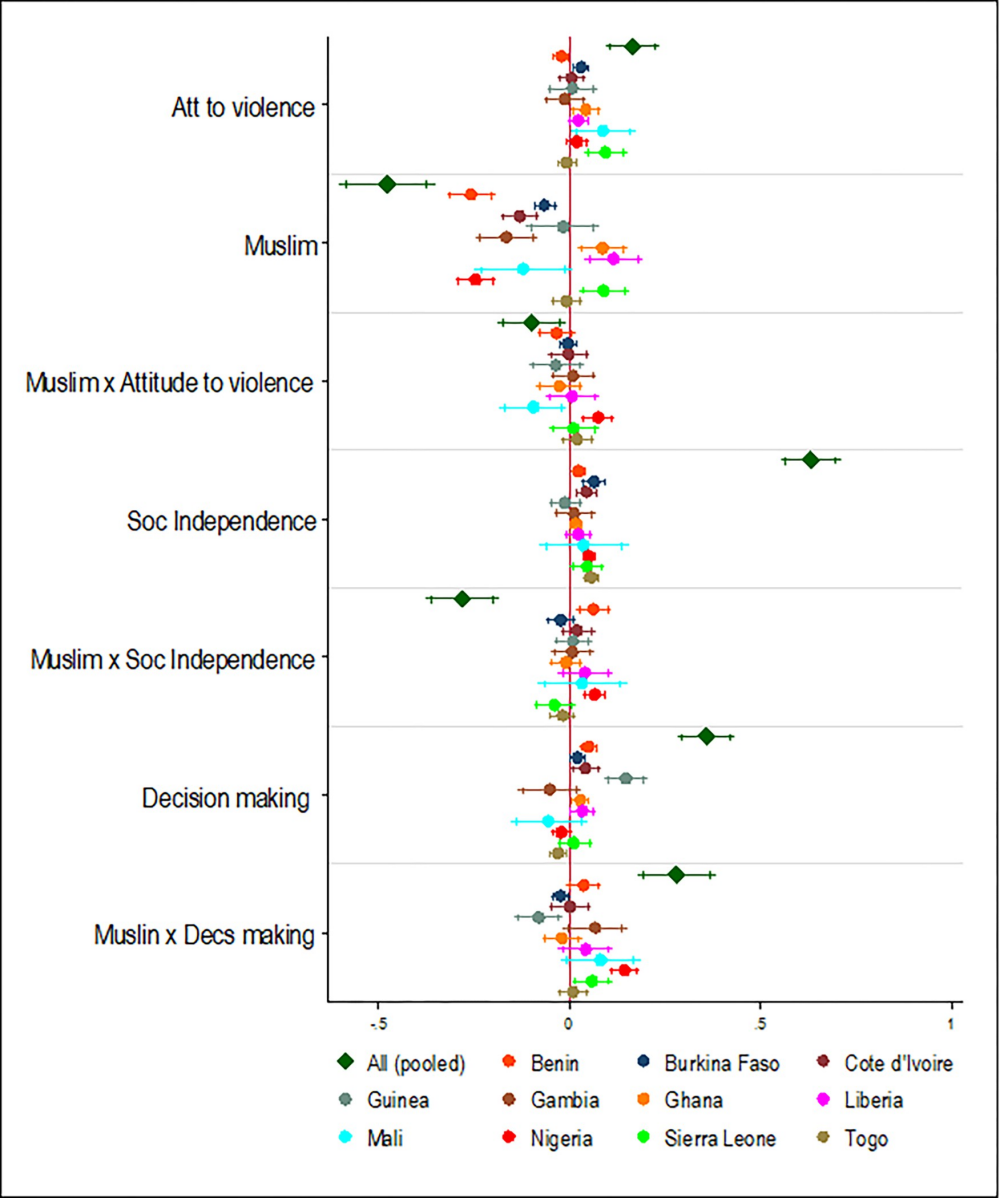
Interaction effects between SWPER domains, religion, and uptake of ANC services. Note: Country dummy variable included in pooled analyses; economic and demographic control variables included in each model; spikes indicate 90% and 95% confidence intervals.

**Fig 11:**
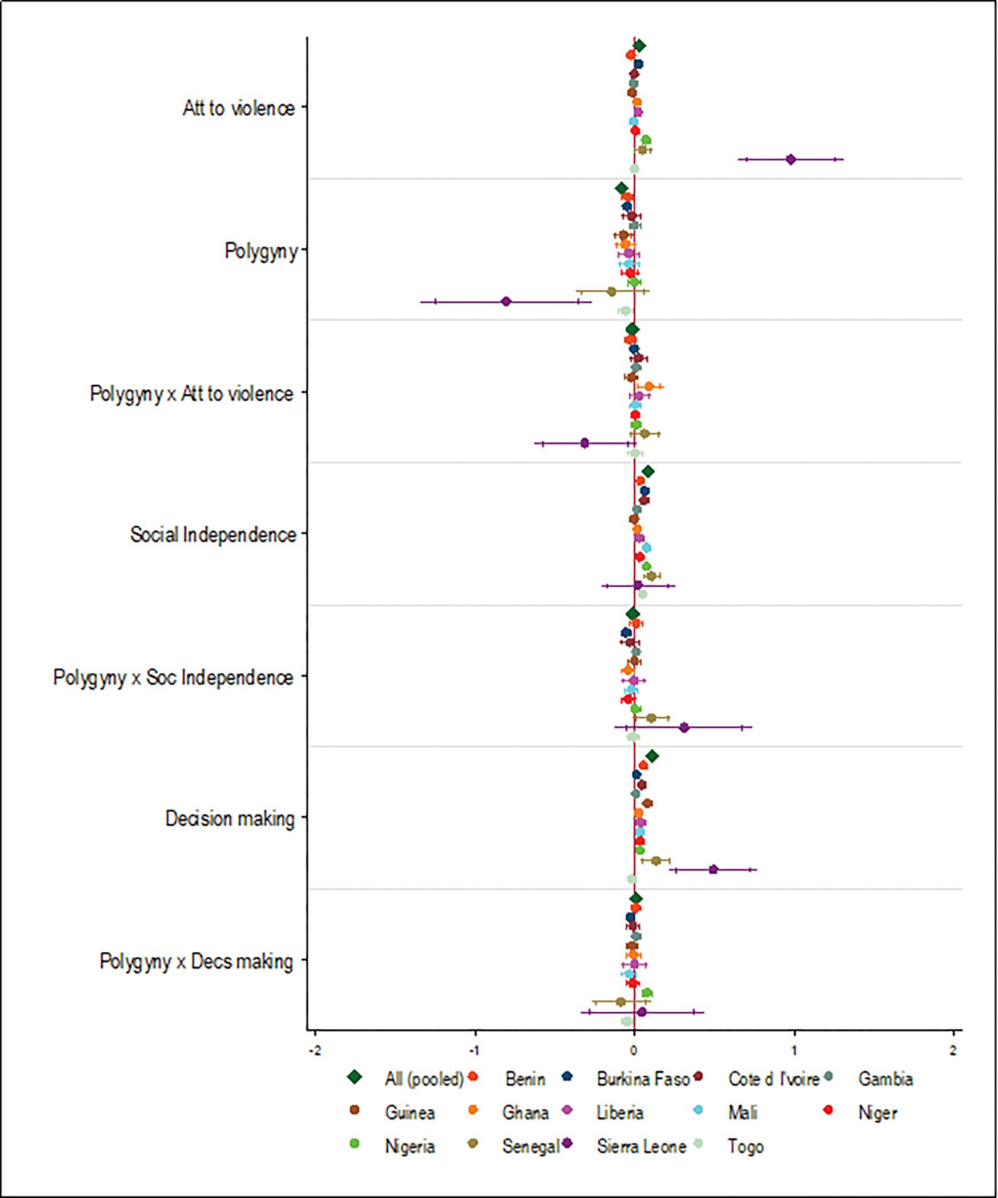
Interaction effects between SWPER domains, marriage type, and uptake of ANC services. Note: Country dummy variable included in pooled analyses; economic and demographic control variables included in each model; spikes indicate 90% and 95% confidence intervals.

A unit increase in the SD of decision-making score for women who identified as Muslims was associated with a 16%-point increase in the likelihood of them attending more ANC services during their last pregnancy (Fig 10). This significant association was also observed in only Guinea, Nigeria, and Sierra Leone. Although not significant in the pooled analyses, a unit increase in SD of decision-making for women who resided in polygynous household was associated with an 8%-point increase in the likelihood of them attending more ANC services in Nigeria (Fig 11).

## Discussions

The present study adds to a limited literature and the study findings are important in the discourse of what key factors within households influence the relationship between women’s empowerment and healthcare utilization for women of reproductive age. Since we defined contextual factors as the cultural and social circumstances within which couple relationships exist, our findings of a relationship between religion, marriage type, women’s empowerment, and uptake of ANC services adds to this limited literature. According to the SWPER index, in West Africa, women in monogamous households appear to be more empowered than their polygynous counterparts and the magnitude of disempowerment appear to be larger for Muslim women relative to Christian women.

While polygyny and Islam appear to be more consistently associated with reduced empowerment in the West African region, in the context of uptake of ANC, the main and interaction effects are less consistent. This suggests that even in situations where prevailing norms and practices broadly limit women’s empowerment, the relationship between empowering women in the three SWPER domains and uptake of ANC services are not clear-cut. Although the association between women’s empowerment and uptake of ANC visits varies across countries in magnitude and statistical significance, the associations are strong enough to suggest the presence of a relationship. This association is consistent with literature from Southeast Asia [49], north Africa, west, and central Africa [50, 51], and eastern Europe [52, 53].The comparability of women’s empowerment studies are limited due to the diverse nature of empowerment measures, indicators, and domains where about 220 unique indicators have been identified [16] and variations exist due to the different approaches used in operationalising women’s empowerment across empowerment measures [7, 8, 54]. While this limitation exists, the present study finding of linkages between women’s empowerment in attitude towards violence, social independence, and decision-making, and uptake of ANC visits are consistent with literature from South-east Asia [49], Nigeria [55], and Kenya [56].

The evidence of a relationship between religion and ANC visits is somewhat mixed. A systematic review of 28 studies found that Muslim women were more likely to seek more ANC visits in India but not in Nigeria and both Muslims and Orthodox Christian faiths were more likely to report increased ANC visits in Ethiopia [4]. This further shed light on the complex nature of the relationship between contextual factors and uptake of healthcare services.

Since evidence suggests that empowering women across the three SWPER domains is beneficial towards improved ANC visits addressing underlying norms and attitudes that encourage the acceptance of violence towards women, restrict women’s social independence and decision-making power is important towards improved health care use. Since decision-making measures women’s agency and autonomy in making choices for themselves in healthcare use, financial expenditure, and social mobility, closing the gender gap especially for polygynous and Muslim women appear to be important towards better uptake of ANC services for women. Further, efforts to empower women should still be context-specific due to existing heterogeneity in the association between social norms and contextual factors and women’s empowerment in different settings.

The SWPER index like other indexes developed using the DHS data is restricted in the types of empowerment indicators and domains it can measure. Perhaps empowerment indicators including autonomy and leisure might be important for women’s uptake of ANC visits. In addition, we did not examine the position of the index woman within polygynous households, and this might be an important determinant of empowerment and access to healthcare services. Since evidence suggests that different religious practices within the Christian faith might have different effects on women’s access to healthcare products and services, a disaggregated analysis might potentially shed more light on the religion-empowerment effects. Our study examines associations hence we cannot claim causation in the identified links between religion, marriage type, women’s empowerment, and uptake of ANC services. This should be an area for future studies.

### Conclusion

The effects of contextual factors on women’s empowerment and uptake of healthcare services have been alluded to but there is limited empirical evidence of the existence of these effects. Contextual factors including prevailing sociocultural practices are broad and varied. Even in regions with similar practices, there are still no clear-cut associations and interactions between women’s empowerment in attitude towards violence, social independence, and decision-making as measured by the SWPER index and contextual factors, and women’s uptake of healthcare services. The present study tried to examine this empowerment-contextual factors relationship and the findings point to the potential benefit of targeting for empowering women in patriarchal and patrilineal societies where polygyny is prevalent, and the practice of Muslim religion is widespread as this could potentially lead to better healthcare use and outcomes.

Our study findings have implications for policy measures and interventions designed to empower women toward better access to health care services. There is need to ensure that the targeting of such policies and intervention are done in a way to ensure that benefits get to specific populations in need. For instance, in a predominantly polygamous society, policy measures and interventions should likely differ in some respects from those designed for a predominantly monogamous society. The mix of policy measures and interventions is expected to reflect these proportions of the two household types. The significance of our study findings is even more crucial for targeting when country differences are considered. Further, there is need for an in-depth examination within countries, communities, and households to understand how religion and marriage type play interacting and deciding roles in the relationship between women and their male spouses, co-wives, and their broader community. Such appraisal would potentially provide much needed nuanced evidence that could guide in efforts to improve women’s attitude towards violence, social independence, and decision-making power.

## Supporting information

S1 Table Summary statistics. (DOCX)

S2 Table Summary statistics (continued). (DOCX)
