# Supplementary material for: Linkages between women’s empowerment, religion, marriage type, and uptake of antenatal care visits in 13 West African countries
Source: PLOS Glob Public Health. 2023 Jun 20;3(6):e0000406. doi: 10.1371/journal.pgph.0000406 (PMC10281566; doi:10.1371/journal.pgph.0000406)
Supplement: S1 Table — (DOCX) [file pgph.0000406.s001.docx]

S1 Table. Summary statistics

| Total (127,287) | | | | | Benin (2018;N=11,170) | | | | Burkina Faso (2010;N=13,374) | | | | Cote d'Ivoire (2012;N=6,437) | | | | Gambia (2013;N=6,905) | | | |
| --- | --- | --- | --- | --- | --- | --- | --- | --- | --- | --- | --- | --- | --- | --- | --- | --- | --- | --- | --- | --- |
|  | mean | SD | min | max | mean | SD | min | max | mean | SD | min | max | mean | SD | min | max | mean | SD | min | max |
| Monogamy | 0.66 | 0.47 | 0 | 1 | 0.62 | 0.49 | 0 | 1 | 0.58 | 0.49 | 0 | 1 | 0.69 | 0.46 | 0 | 1 | 0.60 | 0.49 | 0 | 1 |
| Polygamy | 0.34 | 0.47 | 0 | 1 | 0.38 | 0.49 | 0 | 1 | 0.42 | 0.49 | 0 | 1 | 0.31 | 0.46 | 0 | 1 | 0.40 | 0.49 | 0 | 1 |
| Christian | 0.33 | 0.47 | 0 | 1 | 0.51 | 0.50 | 0 | 1 | 0.28 | 0.45 | 0 | 1 | 0.36 | 0.48 | 0 | 1 | 0.02 | 0.13 | 0 | 1 |
| Muslim | 0.61 | 0.49 | 0 | 1 | 0.33 | 0.47 | 0 | 1 | 0.62 | 0.48 | 0 | 1 | 0.48 | 0.50 | 0 | 1 | 0.98 | 0.13 | 0 | 1 |
| No of regions | 4.58 | 2.97 | 1 | 14 | 5.94 | 3.59 | 1 | 12 | 6.94 | 3.71 | 1 | 13 | 6.15 | 3.06 | 1 | 11 | 4.69 | 2.21 | 1 | 8 |
| Women's edu | 2.96 | 4.49 | 0 | 20 | 2.17 | 3.66 | 0 | 17 | 1.16 | 2.83 | 0 | 18 | 1.97 | 3.48 | 0 | 18 | 2.91 | 4.40 | 0 | 20 |
| Husband's edu | 4.21 | 5.45 | 0 | 23 | 3.60 | 4.94 | 0 | 17 | 1.45 | 3.28 | 0 | 21 | 3.45 | 4.87 | 0 | 18 | 3.60 | 5.40 | 0 | 23 |
| Women's age | 31.40 | 8.55 | 15 | 49 | 31.28 | 8.34 | 15 | 49 | 31.04 | 8.60 | 15 | 49 | 31.39 | 8.41 | 15 | 49 | 30.33 | 8.41 | 15 | 49 |
| Husband's age | 41.60 | 11.71 | 15 | 95 | 38.62 | 10.90 | 15 | 95 | 42.11 | 12.43 | 18 | 95 | 40.82 | 11.07 | 15 | 95 | 43.16 | 12.15 | 19 | 95 |
| Wealth index | 2.89 | 1.41 | 1 | 5 | 3.01 | 1.43 | 1 | 5 | 3.07 | 1.39 | 1 | 5 | 2.76 | 1.40 | 1 | 5 | 2.76 | 1.37 | 1 | 5 |
| Rural location | 0.67 | 0.47 | 1 | 2 | 0.59 | 0.49 | 1 | 2 | 0.74 | 0.44 | 1 | 2 | 0.64 | 0.48 | 1 | 2 | 0.64 | 0.48 | 1 | 2 |
| Household size | 7.75 | 5.51 | 1 | 75 | 7.42 | 4.89 | 1 | 43 | 7.5 | 1.34 | 1 | 46 | 7.60 | 4.71 | 1 | 36 | 14.28 | 11.20 | 1 | 46 |
| Currently employed | 0.68 | 0.21 | 1 | 2 | 0.82 | 0.11 | 1 | 2 | 0.77 | 0.23 | 1 | 2 | 0.73 | 0.19 | 1 | 2 | 0.49 | 0.09 | 1 | 2 |
| Liberia (2013;N=5,868) | | | | | Mali (2018;N=8,332) | | | | Niger (2012;N=9,474) | | | | Nigeria (2018;N=28,788) | | | | Senegal (2018;N=6,381) | | | |
|  | mean | SD | min | max | mean | SD | min | max | mean | SD | min | max | mean | SD | min | max | mean | SD | min | max |
| Monogamy | 0.84 | 0.37 | 0 | 1 | 0.66 | 0.48 | 0 | 1 | 0.65 | 0.48 | 0 | 1 | 0.70 | 0.46 | 0 | 1 | 0.66 | 0.47 | 0 | 1 |
| Polygamy | 0.16 | 0.37 | 0 | 1 | 0.34 | 0.48 | 0 | 1 | 0.35 | 0.48 | 0 | 1 | 0.30 | 0.46 | 0 | 1 | 0.34 | 0.47 | 0 | 1 |
| Christian | 0.84 | 0.37 | 0 | 1 | 0.02 | 0.14 | 0 | 1 | 0.01 | 0.12. | 0 | 1 | 0.42 | 0.49 | 0 | 1 | 0.02 | 0.14 | 0 | 1 |
| Muslim | 0.13 | 0.34 | 0 | 1 | 0.95 | 0.21 | 0 | 1 | 0.99 | 0.12 | 0 | 1 | 0.57 | 0.50 | 0 | 1 | 0.98 | 0.14 | 0 | 1 |
| No of regions | 3.03 | 1.45 | 1 | 5 | 4.53 | 2.65 | 1 | 9 | 4.70 | 1.96 | 1 | 8 | 3.14 | 1.60 | 1 | 6 | 7.51 | 3.87 | 1 | 14 |
| Women's edu | 2.82 | 3.78 | 0 | 16 | 1.88 | 3.45 | 0 | 17 | 1.16 | 2.83 | 0 | 20 | 5.58 | 5.66 | 0 | 20 | 2.38 | 3.81 | 0 | 19 |
| Husband's edu | 6.41 | 4.88 | 0 | 17 | 2.14 | 4.15 | 0 | 17 | 1.62 | 3.72 | 0 | 20 | 7.29 | 6.03 | 0 | 20 | 2.23 | 4.31 | 0 | 21 |
| Women's age | 32.21 | 8.64 | 15 | 49 | 30.07 | 8.52 | 15 | 49 | 29.78 | 8.45 | 15 | 49 | 31.89 | 8.63 | 15 | 49 | 31.34 | 8.64 | 15 | 49 |
| Husband's age | 38.48 | 10.44 | 15 | 95 | 41.60 | 11.77 | 15 | 95 | 41.06 | 11.35 | 16 | 95 | 42.29 | 11.20 | 15 | 95 | 43.27 | 12.66 | 15 | 95 |
| Wealth index | 2.39 | 1.28 | 1 | 5 | 3.06 | 1.40 | 1 | 5 | 3.24 | 1.44 | 1 | 5 | 2.87 | 1.40 | 1 | 5 | 2.54 | 1.31 | 1 | 5 |
| Rural location | 0.66 | 0.47 | 1 | 2 | 0.72 | 0.45 | 1 | 2 | 0.76 | 0.43 | 1 | 2 | 0.64 | 0.48 | 1 | 2 | 0.68 | 0.47 | 1 | 2 |
| Household size | 6.51 | 3.42 | 1 | 53 | 7.07 | 4.45 | 1 | 45 | 7.30 | 4.11 | 1 | 41 | 6.55 | 3.75 | 1 | 67 | 14.13 | 8.62 | 1 | 51 |
| Currently employed | 0.64 | 0.14 | 1 | 2 | 0.58 | 0.20 | 1 | 2 | 0.24 | 0.03 | 1 | 2 | 0.71 | 0.22 | 1 | 2 | 0.54 | 0.18 | 1 | 2 |
