# Supplementary material for: Linkages between women’s empowerment, religion, marriage type, and uptake of antenatal care visits in 13 West African countries
Source: PLOS Glob Public Health. 2023 Jun 20;3(6):e0000406. doi: 10.1371/journal.pgph.0000406 (PMC10281566; doi:10.1371/journal.pgph.0000406)
Supplement: S2 Table — (DOCX) [file pgph.0000406.s002.docx]

S2 Table. Summary statistics (cont.)

|  | Ghana (2014;N=5,456) | | | | Guinea (2018;N=7,812) | | | | Sierra Leone (2013;N=10,575) | | | | Togo (2014;N=6,358) | | | |
| --- | --- | --- | --- | --- | --- | --- | --- | --- | --- | --- | --- | --- | --- | --- | --- | --- |
|  | mean | SD | min | max | mean | SD | min | max | mean | SD | min | max | mean | SD | min | max |
| Monogamy | 0.81 | 0.39 | 0 | 1 | 0.56 | 0.50 | 0 | 1 | 0.65 | 0.48 | 0 | 1 | 0.66 | 0.47 | 0 | 1 |
| Polygamy | 0.19 | 0.39 | 0 | 1 | 0.44 | 0.50 | 0 | 1 | 0.35 | 0.48 | 0 | 1 | 0.34 | 0.47 | 0 | 1 |
| Christian | 0.72 | 0.45 | 0 | 1 | 0.09 | 0.28 | 0 | 1 | 0.18 | 0.39 | 0 | 1 | 0.50 | 0.50 | 0 | 1 |
| Muslim | 0.21 | 0.41 | 0 | 1 | 0.90 | 0.29 | 0 | 1 | 0.81 | 0.39 | 0 | 1 | 0.20 | 0.40 | 0 | 1 |
| No of regions | 5.61 | 2.86 | 1 | 10 | 4.50 | 2.30 | 1 | 8 | 2.30 | 0.94 | 1 | 4 | 3.55 | 1.80 | 1 | 6 |
| Women's edu | 5.28 | 4.73 | 0 | 18 | 1.66 | 3.75 | 0 | 19 | 2.05 | 3.74 | 0 | 17 | 3.31 | 3.79 | 0 | 17 |
| Husband's edu | 6.99 | 5.28 | 0 | 20 | 2.74 | 5.26 | 0 | 19 | 3.42 | 5.12 | 0 | 19 | 5.60 | 4.85 | 0 | 17 |
| Women's age | 33.61 | 8.03 | 15 | 49 | 31.42 | 8.89 | 15 | 49 | 31.68 | 8.45 | 15 | 49 | 32.40 | 8.11 | 15 | 49 |
| Husband's age | 40.86 | 10.59 | 15 | 95 | 45.16 | 13.32 | 15 | 95 | 41.41 | 11.77 | 15 | 95 | 40.31 | 10.89 | 16 | 95 |
| Wealth index | 2.73 | 1.45 | 1 | 5 | 2.85 | 1.40 | 1 | 5 | 2.94 | 1.39 | 1 | 5 | 2.77 | 1.46 | 1 | 5 |
| Rural location | 1.55 | 0.50 | 1 | 2 | 0.70 | 0.46 | 1 | 2 | 1.68 | 0.47 | 1 | 2 | 0.68 | 0.47 | 1 | 2 |
| Household size | 5.38 | 2.76 | 1 | 25 | 7.67 | 4.07 | 1 | 38 | 7.16 | 3.50 | 1 | 32 | 6.88 | 4.11 | 1 | 46 |
| Currently employed | 0.86 | 0.25 | 1 | 2 | 0.70 | 0.12 | 1 | 2 | 0.78 | 0.25 | 1 | 2 | 0.82 | 0.25 | 1 | 2 |
